# Differentiating malignant and benign eyelid lesions using deep learning

**DOI:** 10.1038/s41598-023-30699-5

**Published:** 2023-03-13

**Authors:** Min Joung Lee, Min Kyu Yang, Sang In Khwarg, Eun Kyu Oh, Youn Joo Choi, Namju Kim, Hokyung Choung, Chang Won Seo, Yun Jong Ha, Min Ho Cho, Bum-Joo Cho

**Affiliations:** 1 Department of Ophthalmology, Hallym University College of Medicine, Hallym University Sacred Heart Hospital, 22, Gwanpyeong-Ro 170Beon-Gil, Dongan-Gu, Anyang-Si, Gyeonggi-Do 14068 Republic of Korea; 2 Department of Ophthalmology, Asan Medical Center, Seoul, Korea; 3 Department of Ophthalmology, Seoul National University Hospital, Seoul, Korea; 4 Department of Ophthalmology, Seoul National University College of Medicine, Seoul, Korea; 5 S Eye Center, Ansan, Korea; 6 Department of Ophthalmology, Hallym University College of Medicine, Kangdong Sacred Heart Hospital, Seoul, Korea; 7 Department of Ophthalmology, Seoul National University Bundang Hospital, Seongnam, Korea; 8 Department of Ophthalmology, Seoul Metropolitan Government-Seoul National University Boramae Medical Center, Seoul, Korea; 9 Medical Artificial Intelligence Center, Hallym University Medical Center, Anyang, Korea

**Keywords:** Pathology, Eye cancer, Basal cell carcinoma, Squamous cell carcinoma, Cancer screening, Cancer imaging

## Abstract

Artificial intelligence as a screening tool for eyelid lesions will be helpful for early diagnosis of eyelid malignancies and proper decision-making. This study aimed to evaluate the performance of a deep learning model in differentiating eyelid lesions using clinical eyelid photographs in comparison with human ophthalmologists. We included 4954 photographs from 928 patients in this retrospective cross-sectional study. Images were classified into three categories: malignant lesion, benign lesion, and no lesion. Two pre-trained convolutional neural network (CNN) models, DenseNet-161 and EfficientNetV2-M architectures, were fine-tuned to classify images into three or two (malignant versus benign) categories. For a ternary classification, the mean diagnostic accuracies of the CNNs were 82.1% and 83.0% using DenseNet-161 and EfficientNetV2-M, respectively, which were inferior to those of the nine clinicians (87.0–89.5%). For the binary classification, the mean accuracies were 87.5% and 92.5% using DenseNet-161 and EfficientNetV2-M models, which was similar to that of the clinicians (85.8–90.0%). The mean AUC of the two CNN models was 0.908 and 0.950, respectively. Gradient-weighted class activation map successfully highlighted the eyelid tumors on clinical photographs. Deep learning models showed a promising performance in discriminating malignant versus benign eyelid lesions on clinical photographs, reaching the level of human observers.

## Introduction

Eyelid lesions are commonly encountered in ophthalmology practice^1^. Although most eyelid lesions are benign, a certain proportion are malignant, with increasing incidence rates occurring in many geographical areas^1–3^. If an eyelid lesion is clinically suspected as a malignant tumor based on the history and morphology, surgical biopsy is required to confirm a pathologic diagnosis. A biopsy is usually requested to oculoplastic specialists owing to concerns about potential scars and eyelid deformities. Although the clinical characteristic features of benign and malignant eyelid lesions are well known, the differential diagnosis of eyelid lesions based on clinical morphologies remains a challenge to ophthalmologists^4–7^.

A convolutional neural network (CNN) is a type of deep learning algorithm that indicates an artificial neural network performing mathematical operations using convolution matrices^8^. It has been designed to better utilize spatial and configurable information from images, and to detect the relevant features with minimal human supervision^9^. Several CNN models have been shown to be successful in object recognition and thus adopted in disease diagnosis for various types of medical images^10,11^. Studies utilizing a CNN have recently reported a level of accuracy equal to that of dermatologists for the automated classification of cutaneous tumors in photographic images with homogenous skin background^12,13^.

There are many promising advantages to the development of an artificial intelligence that can differentiate various types of eyelid lesions. Such intelligence can be used as a screening tool for eyelid lesions by general ophthalmologists and can be helpful in reducing “missed malignancies.” It can also help identify lesions that should be biopsied. However, classifying eyelid lesions using clinical images can be technically challenging for artificial intelligence because of the complex anatomical structure of the eyelid and distinct histopathological epidemiology of the eyelid tumors, and there has been no previous studies in our knowledge. Therefore, this study aimed at the application of a deep learning model that enables the differential classification of eyelid lesions based on clinical eyelid photographs using a CNN. We assessed the performance of a CNN in classifying benign and malignant eyelid lesions and compared the diagnostic performance of a trained CNN with that of human clinicians.

## Methods

### Study participants and data extraction

Patients with eyelid lesions were searched through a retrospective review of electronic medical records using international classification of diseases-10-clinical modification diagnosis codes (Supplementary Table S1) or operation codes at Seoul National University Hospital (SNUH). The diagnosis was annotated based on histopathological or clinical findings. All lesions were initially diagnosed by an experienced oculoplastic surgeon (SIK) based on the clinical characteristics. For biopsied lesions, the diagnosis was annotated based on a histopathological report. If the eyelid lesion had not been biopsied, clinical diagnosis was made based on agreement: Eyelid photographs were reviewed by another experienced oculoplastic surgeon (MJL) at the time of enrollment, and the diagnosis was confirmed when the diagnosis agreed with the initial diagnosis.

Eyelid or whole face photographs of these patients, which were taken from October 2004 to April 2020, were included in this study. Photographs were retrieved from the SNUH photograph database in JPEG format with a minimum pixel resolution of 1183 × 690. Poor-quality photographs, photographs inadequate for clinical diagnosis, and postoperative photographs were excluded from the database. When the clinical diagnosis was inconsistent between the two oculoplastic surgeons, the cases were also excluded. In cases with whole face images, two eyelid photos were created by cropping a square with each eyelid separately. For patients with unilateral eyelid lesions, images of the contralateral eyelid photograph without any significant eyelid lesions were annotated as “no lesion” photographs. This study was approved by the Institutional Review Boards of SNUH (No. 1805-175-949) and Hallym University Sacred Heart Hospital (No. 2020-03-026). The protocol of this study adhered to the tenets of the Declaration of Helsinki. Informed consent was waived by the Institutional Review Boards of Seoul National University Hospital, in view of the retrospective nature of the study and the de-identification of patients’ data. Signed statements of informed consent to publish patient photographs were obtained from all identifiable persons.

### Dataset construction

All eyelid photos were classified into three categories based on a histopathologic diagnosis or a clinical diagnosis with expert agreement: malignant lesions, benign lesions, and no lesion categories. The entire dataset was divided into a training dataset and a test dataset with a ratio of 9:1 by random sampling. Dataset splitting was conducted for each class. Random selection was conducted using the patient ID as a key to avoid the simultaneous inclusion of the same patient’s image in the test and training datasets for each class. The training dataset was then further divided into a proper training dataset and a tuning dataset for training with a ratio of 8:1. In the test dataset, only one image was randomly selected per one patient for each class. This proper training/tuning split was applied three times, and the CNN model was trained and evaluated three times independently, using three different training and tuning datasets.

### Image preprocessing and data augmentation

All images were resized into a single size format with a pixel resolution of 690 × 460 and then normalized using the means ([0.485, 0.456, 0.406]) and standard deviation ([0.229, 0.224, 0.225]) of the ImageNet Dataset^14^. A contrast enhancement was applied using contrast-limited adaptive histogram equalization^15–17^ in all channels of the image. The numbers of images in the malignant lesion and no lesion classes of the training dataset were much smaller than those of the benign lesion class training dataset, and thus the images in the malignant lesion and no lesion classes of the training dataset were augmented four times by zooming-in at 5%, 10%, and 20%. The entire training dataset was then further augmented twice through horizontal flipping (Supplementary Fig. S1). The Python libraries opencv (version 4.1.2) and imgaug (version 0.4.0; available at https://github.com/aleju/imgaug; accessed on November 3, 2020) were used for image preprocessing and augmentation.

### CNN model construction

We designed two types of eyelid image classification: a ternary classification (malignant lesion versus benign lesion versus no lesion) and a binary classification (malignant lesion versus benign lesion). For each design, two different CNN architectures, DenseNet-161 and EfficientNetV2-M, were adopted: the former as a widely-used model for medical images and the latter as a state-of-the-art model. The detailed features of the CNN architectures are described elsewhere^18–20^. Briefly, DenseNet is characterized by Denseblock, which concatenates the feature map of the previous layers^18^. EfficientNetV2 finds the optimal CNN architecture using neural architecture search like EfficientNet, and uses progressive learning which changes augmentation magnitude based on the image size^19,20^. The pre-trained models were downloaded from the pytorch website, and the links are as follows: https://pytorch.org/vision/stable/models/generated/torchvision.models.densenet161.html#torchvision.models.DenseNet161_Weights and https://pytorch.org/vision/stable/models/generated/torchvision.models.efficientnet_v2_m.html#torchvision.models.EfficientNet_V2_M_Weights (accessed on Aug 15, 2022).

DenseNet-161 and EfficientNetV2-M were pre-trained with the ImageNet dataset and fine-tuned unfixing the weights. We set all layers unfixed, so every layer was fine-tuned. Categorical cross-entropy was used as the loss function, and the Adam optimizer was applied^21^. The batch size was 3 because it was the maximum batch size that the GPU memory of our server could handle working with EfficientNetV2 model. The learning rate was initially 1e−4, and then reduced by multiplying by 0.1 every 10 epochs until the learning rate reached 1e−7. We adopted early stopping after the 30th epoch with a patience value of 30 according to the loss for the tuning dataset, or the validation loss value. In the training process, at epochs when the validation loss value was greater than the minimum validation loss so far, the model was not updated. Thus, the model that was saved at the epoch showing the minimum validation loss in the training stage was selected as the final model to prevent overfitting. The training server was implemented with six NVIDIA GTX 1080ti graphic processing units, dual Intel Xeon E5-2690 central processing units, 128 GB RAM, and a customized water-cooling system.

### Comparison of the diagnostic performance between CNN and clinicians

After constructing the CNN models using the training dataset, the diagnostic performance of the models was evaluated using the test set. The main outcomes were the discrimination performance of the established CNN models for ternary or binary classification.

The diagnostic performances of the established CNN models and nine clinicians were compared. A panel of human clinicians was constructed, including three oculoplastic specialists, three board-certified ophthalmologists, and three ophthalmology residents.

### Saliency map

To visualize the pixels of interest, a saliency map was created using a gradient-weighted class activation map (Grad-CAM). Grad-CAM uses the gradient information flowing into the last convolutional layer and is applicable without altering the CNN architecture^22^. It produces a localization heatmap overlapping the existing image, and its visualization outperforms previous approaches on interpretability and faithfulness to the original model.

### Statistical analysis

The area under the receiver operating characteristic curve (AUC) of the CNN model was calculated and compared using the DeLong test. In addition, the sensitivity, specificity, positive predictive value, and negative predictive value for a binary classification were calculated at the point having Youden’s J statistic maximized. Analyses were conducted using IBM SPSS Statistics version 24.0. (IBM Co., New York, USA) and MedCalc version 19.0.4 (MedCalc Software Ltd., Ostend, Belgium).

### Conference presentation

Presented as an e-poster at the American Academy of Ophthalmology 2020 Virtual Meeting, November 2020.

Presented as an oral presentation at the American Society of Ophthalmic Plastic and Reconstructive Surgery 51st Annual Fall Scientific Symposium.

## Results

**Figure 1:**
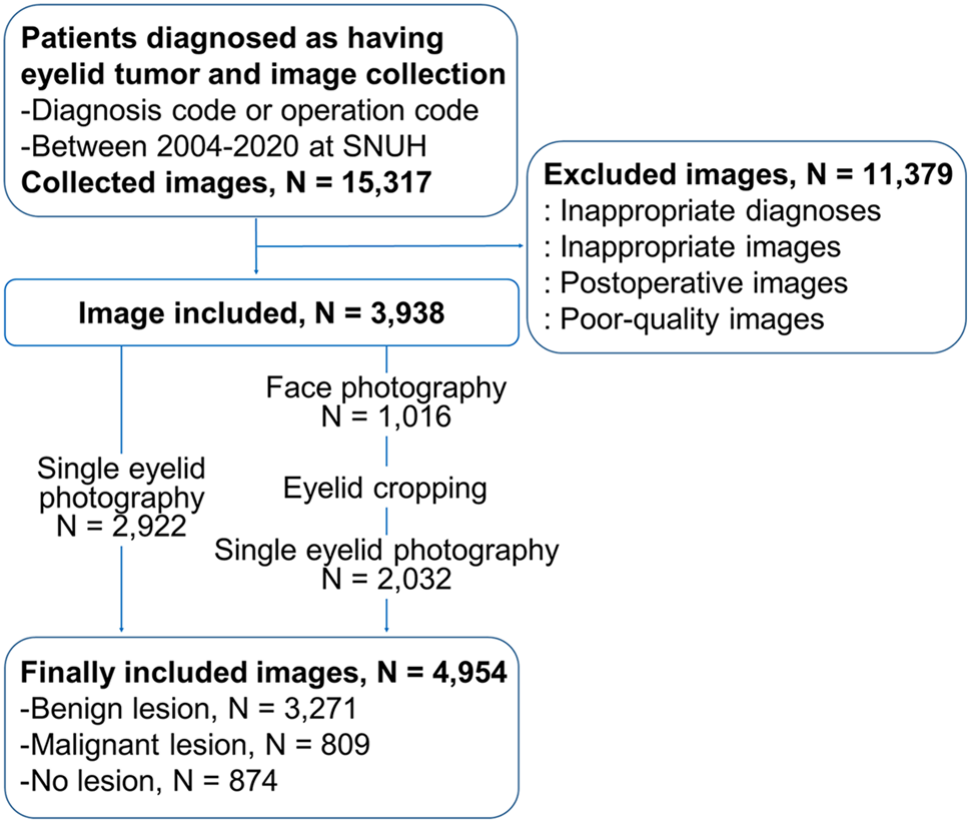
Flow diagram of eyelid image dataset construction.

A flow diagram and compositions of the constructed image dataset from the 928 patients are shown in Fig. 1 and Table 1, respectively. The most common diagnosis in the malignant lesion category was basal cell carcinoma (N = 306, 37.8%), followed by sebaceous gland carcinoma (N = 287, 35.5%). The most common diagnosis in the benign lesion category was chalazion (N = 1377, 42.1%), followed by nevus (N = 784, 24.0%). After dataset augmentation, the training dataset is composed 5200 images in the malignant lesion category, 5,608 images in the benign lesion category, and 5611 images in the no-lesion category. The ratio of each class was 1:4:1 originally, and it was changed to 1:1:1 after balancing the training dataset. The performance plateaued after approximately 20 training epochs. The inference time for ternary classification per one image in the test dataset was 0.0119 ± 0.0001 s by DenseNet-161 and 0.0117 ± 0.0004 by EfficientNetV2-M. The inference time for binary classification per one image in the test dataset was 0.0111 ± 0.0001 s by DenseNet-161 and 0.0110 ± 0.0003 by EfficientNetV2-M. Learning curves of the CNN models for ternary and binary classifications are presented in Supplementary Fig. S2.

**Table 1:** Data composition of the constructed eyelid photograph dataset.

	Whole dataset		Training dataset		Test dataset	
	Images, N	Patients, N	Images, N	Patients, N	Images, N	Patients, N
Overall	4954	928	4475	883	134	134
Malignant lesion	809	142	727	128	14	14
Benign lesion	3271	804	2953	724	80	80
No lesion	874	503	795	453	50	50

### Lesion differentiation performance for ternary classification (malignant lesion versus benign lesion versus no lesion)

The mean overall diagnostic accuracy of deep learning models for the ternary classification was 82.1% (95% CI 81.2–82.9%) when using DenseNet-161, and 83.0% (95% CI 81.8–84.1%) when applying EfficientNetV2-M, respectively. Meanwhile, the mean overall accuracies of the ophthalmology residents, board-certified ophthalmologists, and oculoplastic specialists were 87.7% (95% CI 86.8–88.5%), 87.0% (95% CI 86.7–87.2%), and 89.5% (95% CI 82.1–96.8%), respectively.

The per-category diagnostic performance of the deep learning models is presented in Table 2. The diagnostic accuracies were highest in differentiating the malignant lesions. The per-class diagnostic AUCs for malignant lesions were 0.960 (95% CI 0.956–0.963) for DenseNet-161 and 0.955 (95% CI 0.950–0.959) for EfficientNetV2-M, respectively.

**Table 2:** Per-class diagnostic performances of deep-learning models differentiating malignant lesion, benign lesion, and no lesion categories.

Model	Class	Diagnostic performance, % (95% CI)					AUC (95% CI)
		Accuracy	Sensitivity	Specificity	PPV	NPV	
DenseNet-161	Malignant lesion	92.8 (92.2–93.3)	83.3 (82.6–84.0)	93.8 (93.1–94.5)	62.0 (59.7–64.3)	98.1 (98.0–98.1)	0.960 (0.956–0.963)
	Benign lesion	82.8 (82.0–83.7)	83.3 (83.2–84.2)	81.7 (80.5–82.9)	85.2 (84.3–86.1)	80.0 (79.2–80.7)	0.907 (0.902–0.911)
	No lesion	88.6 (88.3–88.9)	79.3 (77.6–81.0)	93.6 (93.1–94.0)	87.3 (86.6–87.9)	89.7 (89.0–90.4)	0.962 (0.960–0.963)
EfficientNetV2-M	Malignant lesion	92.1 (91.2–92.9)	78.5 (77.4–79.7)	93.5 (92.5–94.6)	63.3 (59.9–66.6)	97.6 (97.5–97.7)	0.955 (0.950–0.959)
	Benign lesion	84.2 (83.2–85.2)	87.9 (86.5–89.2)	79.6 (79.0–80.3)	84.3 (83.7–84.9)	84.5 (82.9–86.1)	0.922 (0.913–0.930)
	No lesion	89.8 (89.1–90.4)	76.7 (74.8–78.4)	96.8 (96.1–97.4)	93.2 (92.1–94.4)	88.8 (88.0–89.5)	0.965 (0.963–0.966)

*AUC* area under the receiver operating characteristic curve, *CI* confidence interval, *NPV* negative predictive value, *PPV* positive predictive value.

### lesion differentiation performances for binary classification (malignant lesion versus benign lesion)

**Figure 2:**
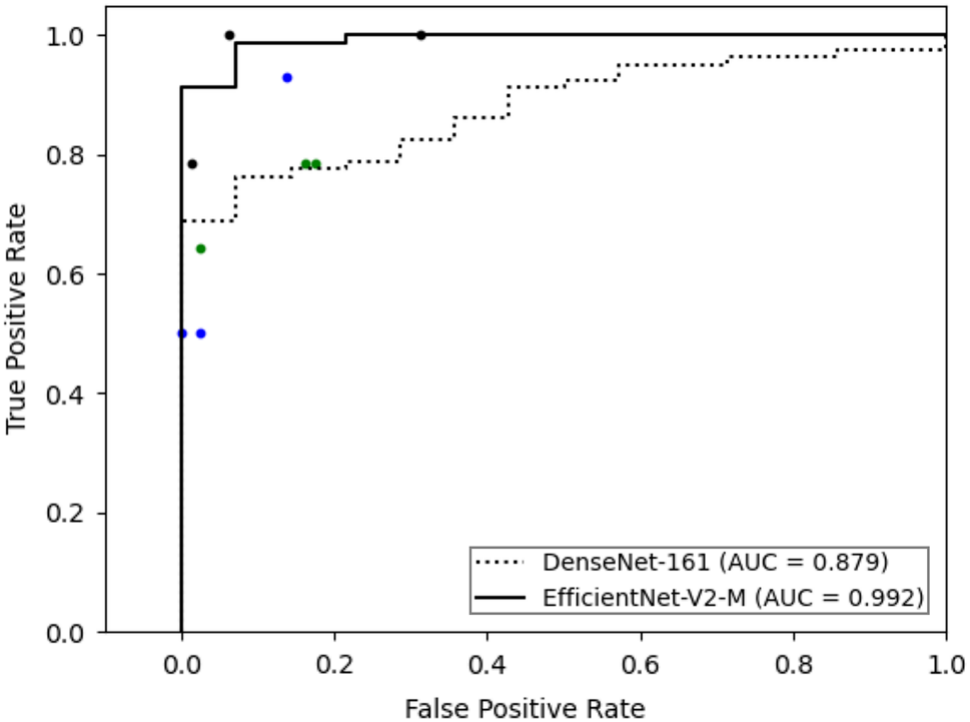
Receiver operating characteristic curves of deep-learning models in differentiating malignant and benign eyelid lesions. The diagnostic performances of nine ophthalmology clinicians on the same test set are shown by single dots. Black dots indicate the diagnostic performances of oculoplastic specialists, blue dots are those of board-certified general ophthalmologists, and the green dots are those of ophthalmology residents.

For binary classification, we use a ratio of 1:1 for images containing malignant lesion and benign lesion. The performances of deep learning models used for differentiating malignant and benign eyelid lesions are presented in Table 3. EfficientNetV2-M showed a better performance (AUC 0.950; 95% CI 0.942–0.957) than DenseNet-161 (AUC 0.908; 95% CI 0.899–0.916). The mean diagnostic accuracy, sensitivity, and specificity of EfficientNetV2-M were 92.5% (95% CI 91.8–93.2%), 90.4% (95% CI 88.6–92.1%), and 87.8% (95% CI 85.7–89.8%), respectively. The mean overall accuracy of the clinicians was 87.9% (95% CI 85.3–90.4%) by the oculoplastic specialists, 90.0% (95% CI 89.5–90.6%) by the general ophthalmologists, and 85.8% (95% CI 84.6–87.0%) by the ophthalmology residents, respectively (Table 3 and Fig. 2). The trained models showed a higher sensitivity (90.4% and 92.8%) than general ophthalmologists or ophthalmology residents (64.2% and 73.8%), achieving the oculoplastic specialists’ level (92.8%). Regarding the receiver operating characteristics, the performance of EfficientNetV2-M was superior to those of ophthalmology residents.

**Table 3:** Diagnostic performances of deep-learning models and human clinicians in differentiating malignant and benign eyelid lesions.

Model	Diagnostic performance, % (95% CI)					AUC (95% CI)
	Accuracy	Sensitivity	Specificity	PPV	NPV	
DenseNet-161	87.5 (86.6–88.4)	92.8 (91.6–93.9)	77.4 (76.8–77.9)	42.0 (41.5–42.4)	98.4 (98.1–98.6)	0.908 (0.899–0.916)
EfficientNetV2-M	92.5 (91.8–93.2)	90.4 (88.6–92.1)	87.8 (85.7–89.8)	63.7 (59.8–67.5)	98.2 (97.8–98.5)	0.950 (0.942–0.957)
Oculoplasty specialists	87.9 (85.3–90.4)	92.8 (90.3–95.3)	87.0 (83.8–90.3)	67.0 (61.3–72.8)	98.7 (98.3–99.2)	–
Board-certified ophthalmologists	90.0 (89.5–90.6)	64.2 (59.2–69.2)	94.5 (93.1–96.0)	77.3 (72.6–81.9)	94.0 (93.3–94.8.)	–
Ophthalmology residents	85.8 (84.6–87.0)	73.8 (72.1–75.4)	87.9 (86.2–89.5)	57.2 (52.9–61.5)	95.1 (94.9–95.3)	–

*AUC* area under the receiver operating characteristic curve, *CI* confidence interval, *NPV* negative predictive value, *PPV* positive predictive value.

### Saliency map

**Figure 3:**
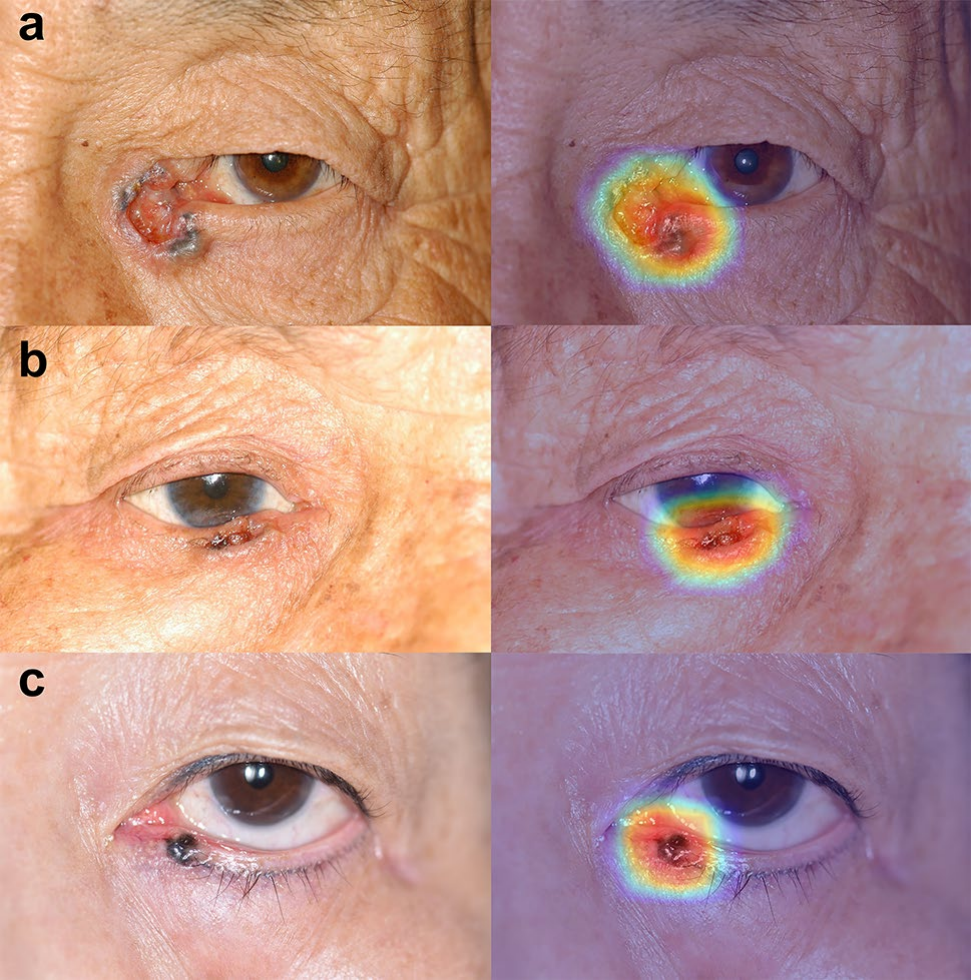
Representative gradient-weighted class activation mapping images of the EfficientNetV2-M model. In differentiating malignant (**a**–**c**) eyelid lesions, figures are shown in pairs of the original eyelid image (left) and corresponding activation mapping image (right).

Representative Grad-CAM images of the EfficientNetV2-M model for binary classification differentiating malignant and benign eyelid lesions are shown in Fig. 3. The saliency maps highlighted regions that the CNN likely focused on when predicting malignant lesions. In general, the activated regions corresponded well with the tumor location.

## Discussion

In this study, the CNN-based deep learning models adopting the DenseNet-161 and EfficientNetV2-M architectures showed an excellent performance in terms of ternary and binary classifications for differentiating malignant and benign eyelid lesions. In the binary classification, the diagnostic accuracies of these deep learning models for differentiating malignant lesions were similar to those of oculoplasty specialists. In the ternary classification, the overall diagnostic accuracy of the CNNs were slightly lower than those of the clinicians. However, the diagnostic performance was highest in differentiating the malignant lesion category.

In 2017, Esteva et al.^12^ reported the performance of deep neural networks in skin cancer classification using photographic and dermoscopic images, suggesting the possibilities of the novel use of deep learning in the dermatologic field. After this landmark publication, several published reports have confirmed that the diagnostic performance of the CNN-based methods outperformed dermatologists in classifying skin cancer^13,23–25^. However, most of these studies investigated skin tumors on the background of normal homogeneous skin. The differentiation of lesions at specific anatomic sites has rarely been implemented. Cho et al.^26^ investigated the performance of a trained CNN in classifying benign and malignant lip diseases and reported a similar performance as board-certified dermatologists. We believe that this is the first study to investigate the use of deep learning models to differentiate eyelid lesions. Haenssle et al.^27^ recently compared the diagnostic performance of a CNN against dermatologists for face and scalp lesions and reported that the CNN showed a higher sensitivity (96.2% versus 84.2%) when fixing the specificity of the CNN at the mean specificity of the dermatologists (69.4%). There were only four cases of eyelid lesions among the 100 cases in their test set.

The surface anatomy of the eyelid is complicated and contains various structures, including the eyebrow, eyelid crease, eyelashes, and meibomian glands. Eyelid images also frequently contain ocular surface areas such as the cornea, part of the bulbar conjunctiva, semilunar fold, and caruncle, and there are numerous individual variations. Therefore, it is technically challenging for deep learning algorithms to classify eyelid lesions using clinical images. Despite this structural complexity and tricky background, the accuracy of binary classification was 90.0–92.5%, and the accuracy of ternary classification reached 82.1–83.0% when using the deep learning model. The diagnostic accuracies of the EfficientNet-V2-M was superior to those of the clinicians in terms of binary classification, differentiating malignant from benign eyelid lesions.

We tested two deep learning models, i.e., ternary and binary classification models. The binary classification models were devised with the goal of developing an assistant tool for clinicians, and we applied the ternary classification models for the exploitation of automated screening tools or diagnostic applications. In this study, the performances of the CNN-based approaches were different between ternary and binary classifications and were not uniform across the categories. The binary classification showed a higher accuracy than the ternary classification. However, the differentiation of the malignant lesion category showed the highest accuracy (92.8% with DenseNet-161 and 92.1% with EfficientNetV2-M) for the ternary classification models. We hypothesized that the less distinct appearance of benign lesions and complicated backgrounds made it difficult for a CNN to classify the benign lesion and no lesion categories. It is well known that the accuracy of a CNN tends to decline as the number of classes increases^28^. A larger number of training images are needed to improve the accuracy of the ternary classification and develop a deep learning algorithm that can diagnose specific eyelid diseases.

In this study, the diagnostic performance of EfficientNetV2-M-based deep learning models was higher than that of DenseNet-161-based deep learning models, especially in the binary classification. It is possible that EfficientNetV2-M architecture would be more suitable for recognizing the characteristics of eyelids than DenseNet-161. A previous dermatologic study on the classification of common skin conditions reported that the DenseNet architecture provides better results than other contemporary architectures^29^. Problems related to over-fitting may occur in more complex CNN models. Instead of drawing representational power from extremely complex architectures, the DenseNet architecture pursues shorter connections between layers close to the input and those close to the output^18^. Nevertheless, the EfficientNetV2, the state-of-the-art architecture, showed a better performance in this study. EfficientNetV2 was developed in 2019 and known to have faeter training speed and better efficienty than previous models. The superiority of EfficientNetV2 over other CNN models in terms of differentiating eyelid lesions should be repeatedly verified in the future studies.

The clinical diagnosis of eyelid lesions is entirely based on a detailed history and gross morphology. It depends on the experience of the clinician, and there are no imaging modalities or ancillary tests. Although the morphological characteristics of malignant eyelid lesions are well known, differentiating between benign and malignant eyelid lesions is occasionally difficult. Basal cell carcinoma can be confused with nevus, papilloma, and hydrocystoma^4^. Sebaceous gland carcinoma is commonly misdiagnosed as a benign tumor or inflammatory lesion, including chalazion or blepharoconjunctivitis, resulting in a significant diagnostic delay^6^. Early diagnosis of malignant eyelid disorders is important for successful treatment and a better prognosis. In addition, higher T staging increases the risk of regional lymph node metastasis and tumor-related death in sebaceous gland carcinoma^30^. Moreover, eyelid reconstruction is technically more difficult when the defect is large and involves canthal structures. In this study, the promising accuracy of a CNN in binary classification, comparable with that of the clinicians, suggests that CNNs have the potential for application under clinical settings. Clinicians may be able to improve the accuracy of their clinical diagnoses with the aid of a CNN. In addition, CNNs classify the lesions based solely on images without any clinical information, including age, duration of the lesion, or anatomical site. These data are easily available and may help enhance the performance of CNNs.

This study has several limitations. First, most of the images used were obtained from Korean subjects, and the performance of our deep learning models should be validated using independent datasets from different ethnic populations. Second, histopathological diagnosis or clinical diagnosis was annotated to the images as a gold standard, and there is a possibility that a clinical diagnosis will be incorrect even though two experienced oculoplastic surgeons agree on the diagnosis. Third, the number of images in the training set was insufficient, and the proportion of each group was unequal. We therefore manipulated the training set, augmenting the number of images, and bias from the image augmentation is possible. Fourth, although the saliency maps highlighted regions of interest, the mechanism of the CNN underlying the classification of the eyelid lesions remains unclear. Further research with technical aspects that explore the mechanisms underlying such classification is needed. Finally, as can be inferred from comparable accuracies among the three groups of clinicians, our dataset may contain a significant number of advanced malignant lesions. If the CNN models are constructed with a dataset containing a larger number of early-stage malignant lesions and maintain their promising accuracies, they will have more clinical significance as a screening tool.

In conclusion, we applied deep learning algorithms for classifying eyelid lesions using clinical photographs, and the diagnostic performances of deep learning models differentiating malignant lesions from benign lesions were found to be comparable to those of human ophthalmologists. The results of this study suggest that our deep learning models may assist clinicians in differentiating malignant eyelid lesions, enabling an early diagnosis and improving the clinical outcomes. Further validation of these algorithms in a community setting is needed.

## Supplementary Information

Supplementary Information.

## Data Availability

The datasets generated during and/or analyzed during the current study are available from the corresponding authors on reasonable request.

## References

[1] Lin HY, Cheng CY, Hsu WM, Kao WH, Chou P (2006). Incidence of eyelid cancers in Taiwan: A 21-year review. Ophthalmology.

[2] Jung SK, Lim J, Yang SW, Jee D, Won YJ (2020). Nationwide trends in the incidence and survival of eyelid skin cancers in Korea. Ophthalmic Epidemiol..

[3] Quigley C (2019). National incidence of eyelid cancer in Ireland (2005–2015). Eye.

[4] Kersten RC, Ewing-Chow D, Kulwin DR, Gallon M (1997). Accuracy of clinical diagnosis of cutaneous eyelid lesions. Ophthalmology.

[5] Margo CE (1999). Eyelid tumors: accuracy of clinical diagnosis. Am. J. Ophthalmol..

[6] Muqit MM (2013). Observational prospective cohort study of patients with newly-diagnosed ocular sebaceous carcinoma. Br. J. Ophthalmol..

[7] Ozdal PC, Codere F, Callejo S, Caissie AL, Burnier MN (2004). Accuracy of the clinical diagnosis of chalazion. Eye.

[8] Albawi, S., Mohammed, T. & Al-Zawi, S. *Understanding of a Convolutional Neural Network*. ieeexplore.ieee.org/document/8308186 (2017).

[9] Shen D, Wu G, Suk H-I (2017). Deep learning in medical image analysis. Annu. Rev. Biomed. Eng..

[10] Russakovsky O (2015). ImageNet large scale visual recognition challenge. Int. J. Comput. Vis..

[11] Krizhevsky A, Sutskever I, Hinton GE (2012). ImageNet classification with deep convolutional neural networks. Adv. Neural Inf. Process. Syst..

[12] Esteva A (2017). Dermatologist-level classification of skin cancer with deep neural networks. Nature.

[13] Han SS (2018). Classification of the clinical images for benign and malignant cutaneous tumors using a deep learning algorithm. J. Invest. Dermatol..

[14] Ngo, Q. T. & Yoon, S. *Weighted-center Loss for Facial Expressions Recognition*. ieeexplore.ieee.org/document/9289472 (2020).

[15] Sahu S, Singh AK, Ghrera SP, Elhoseny M (2019). An approach for de-noising and contrast enhancement of retinal fundus image using CLAHE. Opt. Laser Technol..

[16] Mansour RF, Aljehane NO (2021). An optimal segmentation with deep learning based inception network model for intracranial hemorrhage diagnosis. Neural Comput. Appl..

[17] Ragab M, Albukhari A, Alyami J, Mansour RF (2022). Ensemble deep-learning-enabled clinical decision support system for breast cancer diagnosis and classification on ultrasound images. Biology..

[18] Huang, G., Liu, Z., Van Der Maaten, L. & Weinberger, K. Q. *Densely Connected Convolutional Networks*. http://arxiv.org/abs/1608.06993 (2016).

[19] Tan, M. & Le, Q. V. *EfficientNet: Rethinking Model Scaling for Convolutional Neural Networks*. http://arxiv.org/1905.11946 (2019).

[20] Tan, M. & Le, Q. V. *EfficientNetV2: Smaller Models and Faster Training*. http://arxiv.org/2104.00298 (2021).

[21] Cho BJ (2019). Automated classification of gastric neoplasms in endoscopic images using a convolutional neural network. Endoscopy.

[22] Selvaraju, R. R. *et al.**Grad-CAM: Visual Explanations from Deep Networks via Gradient-Based Localization*. http://arxiv.org/1610.02391 (2017).

[23] Brinker TJ (2019). Deep neural networks are superior to dermatologists in melanoma image classification. Eur. J. Cancer..

[24] Liu Y (2020). A deep learning system for differential diagnosis of skin diseases. Nat. Med..

[25] Winkler JK (2019). Association between surgical skin markings in dermoscopic images and diagnostic performance of a deep learning convolutional neural network for melanoma recognition. JAMA Dermatol..

[26] Cho SI (2020). Dermatologist-level classification of malignant lip diseases using a deep convolutional neural network. Br. J. Dermatol..

[27] Haenssle HA (2021). Skin lesions of face and scalp: Classification by a market-approved convolutional neural network in comparison with 64 dermatologists. Eur. J. Cancer..

[28] Deng J, Berg AC, Li K, Fei-Fei L, Daniilidis K, Maragos P, Paragios N (2010). what does classifying more than 10,000 image categories tell us?. European Conference on Computer Vision.

[29] Pangti R (2021). A machine learning-based, decision support, mobile phone application for diagnosis of common dermatological diseases. J. Eur. Acad. Dermatol. Venereol..

[30] Hsia Y, Yeh CY, Wei YH, Chen LW, Liao SL (2019). Eyelid sebaceous carcinoma: Validation of the 8th edition of the American Joint Committee on cancer T staging system and the prognostic factors for local recurrence, nodal metastasis, and survival. Eye.

