# Supplementary material for: Differentiating malignant and benign eyelid lesions using deep learning
Source: Sci Rep. 2023 Mar 13;13:4103. doi: 10.1038/s41598-023-30699-5 (PMC10011394; doi:10.1038/s41598-023-30699-5)
Supplement: Supplementary file 1 — Supplementary Information. [file 41598_2023_30699_MOESM1_ESM.docx]

**Supplementary Materials for:**

**Differentiating malignant and benign eyelid lesions using deep learning**

**Supplementary Figure S1.** An example showing the image preprocessing and data augmentation process. CLAHE: contrast-limited adaptive histogram equalization


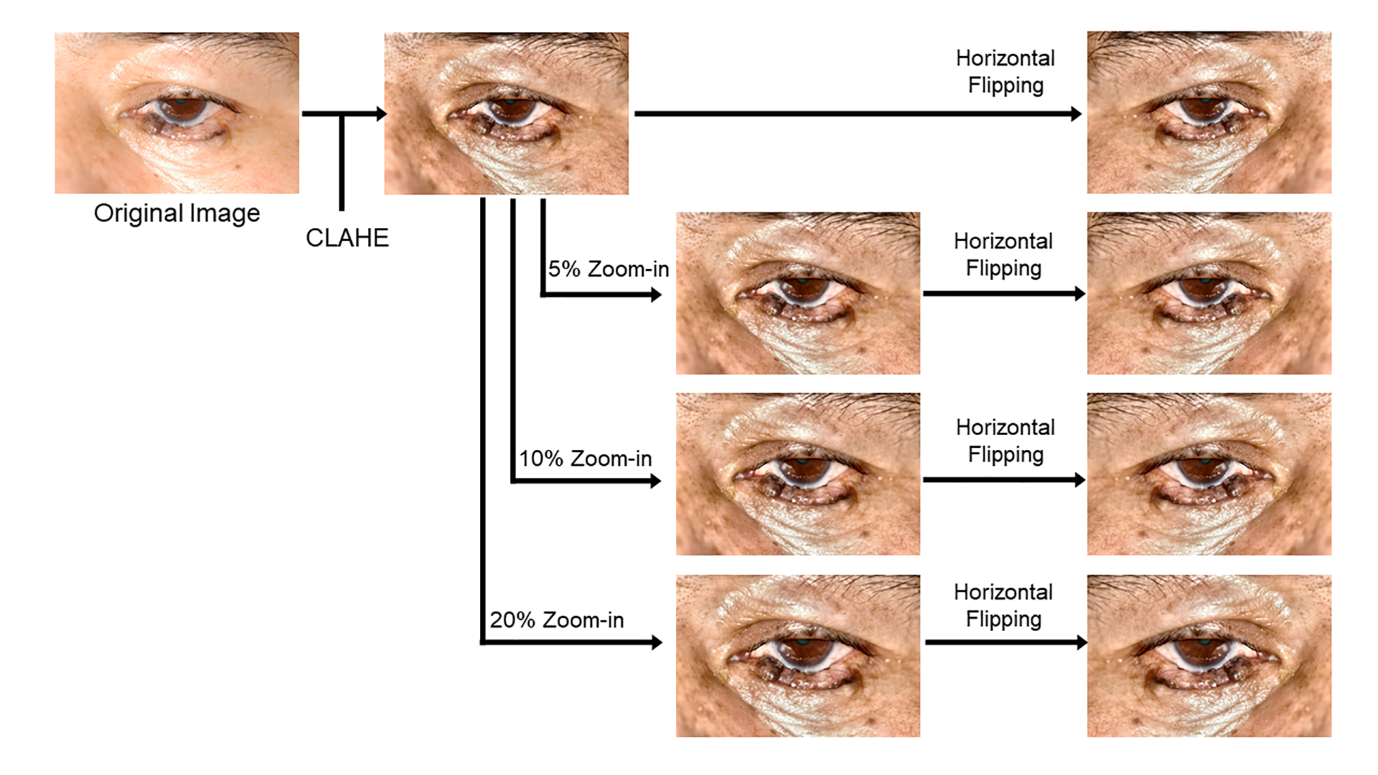


**Supplementary Figure S2.** Learning curves of convolutional neural network models. (a) DenseNet-161 for ternary classification. (b) DenseNet-161 for binary classification. (c) EfficientNetV2-M for ternary classification. (d) EfficientNetV2-M for binary classification.

**
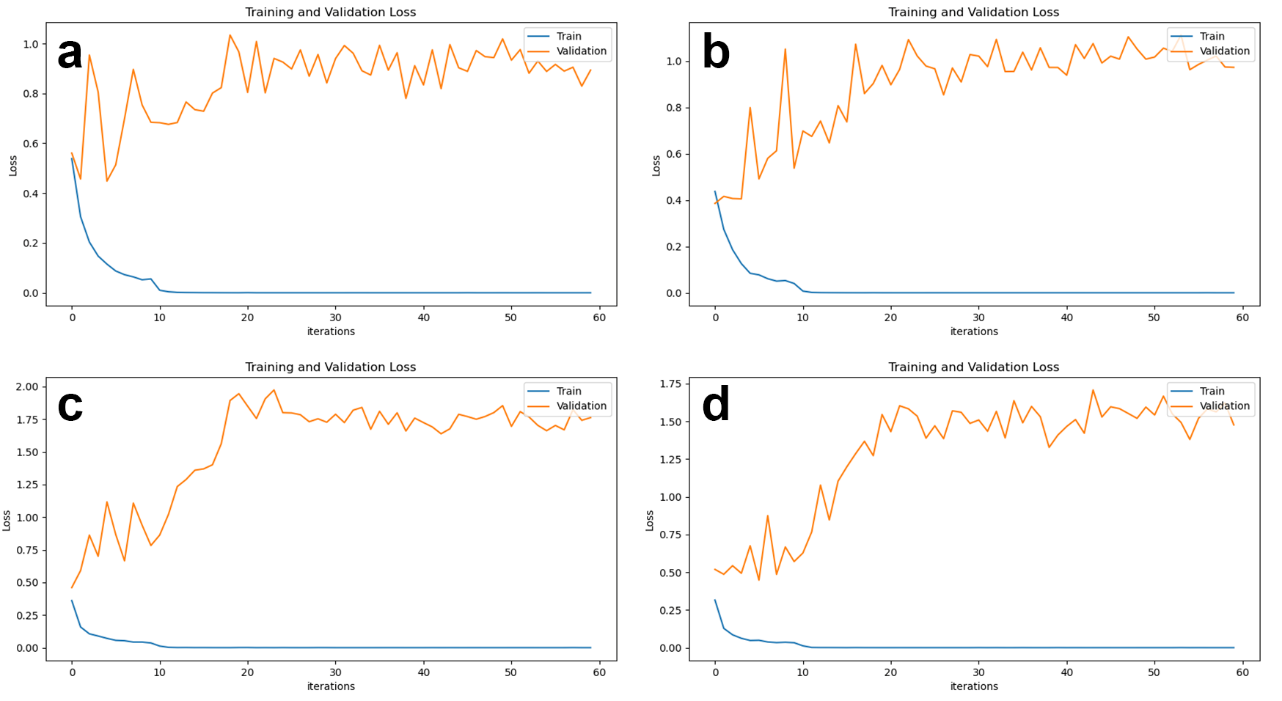
**

**Supplementary Table S1.** International classification of diseases-10-clinical modification (ICD-10-CM) codes of diagnosis used for screening study participants.

| ICD-10-CM | Diagnosis |
| --- | --- |
| C43.1 | Malignant melanoma of eyelid, including canthus |
| C44.1 | Other and unspecified malignant neoplasm of skin of eyelid, including canthus |
| C44.10 | Unspecified malignant neoplasm of skin of eyelid, including canthus |
| C44.11 | Basal cell carcinoma of skin of eyelid, including canthus |
| C44.12 | Squamous cell carcinoma of skin of eyelid, including canthus |
| C44.13 | Sebaceous cell carcinoma of skin of eyelid, including canthus |
| C44.19 | Other specified malignant neoplasm of skin of eyelid, including canthus |
| C4A.10 | Merkel cell carcinoma of eyelid, including canthus |
| C49.0 | Malignant neoplasm of connective and soft tissue of head, face and neck |
| D03.1 | Melanoma in situ of eyelid, including canthus |
| D04.1 | Carcinoma in situ of skin of eyelid, including canthus |
| B07.9 | Viral wart, unspecified |
| D18.01 | Hemangioma of skin and subcutaneous tissue |
| D18.1 | Lymphangioma, any sit |
| D21.0 | Benign neoplasm of connective tissue of eyelid |
| D22.1 | Melanocytic nevi of eyelid, including canthus |
| D23.1 | Other benign neoplasm of skin of eyelid, including canthus |
| D23.30 | Other benign neoplasm of skin of unspecified part of face |
| D23.9 | Other benign neoplasm of skin, unspecified |
| D48.1 | Neoplasm of uncertain behavior of connective tissue of eyelid |
| D48.5 | Neoplasm of uncertain behavior of skin of eyelid |
| H00.1 | Chalazion |
| H01.8 | Other specific inflammations of eyelid |
| H01.9 | Unspecified inflammation of eyelid |
| H02.6 | Xanthelasma of eyelid |
| H02.8 | Other specified disorders of eyelid |
| H02.82 | Cysts of eyelid |
| H02.87 | Vascular anomalies of eyelid |
| H04.11 | Dacryops |
| L57.0 | Actinic keratosis |
| L82.1 | Other seborrheic keratosis |
| Q82.5 | Congenital non-neoplastic nevus |
